# Visible light photooxidative performance of a high-nuclearity molecular bismuth vanadium oxide cluster

**DOI:** 10.3762/bjnano.5.83

**Published:** 2014-05-26

**Authors:** Johannes Tucher, Carsten Streb

**Affiliations:** 1 Ulm University, Institute of Inorganic Chemistry I, Albert-Einstein-Allee 11, 89081 Ulm, Germany

**Keywords:** photocatalysis, photooxidation, polyoxometalate, self-assembly, vanadium oxide

## Abstract

The visible light photooxidative performance of a new high-nuclearity molecular bismuth vanadium oxide cluster, H_3_[{Bi(dmso)_3_}_4_V_13_O_40_], is reported. Photocatalytic activity studies show faster reaction kinetics under anaerobic conditions, suggesting an oxygen-dependent quenching of the photoexcited cluster species. Further mechanistic analysis shows that the reaction proceeds via the intermediate formation of hydroxyl radicals which act as oxidant. Trapping experiments using ethanol as a hydroxyl radical scavenger show significantly decreased photocatalytic substrate oxidation in the presence of EtOH. Photocatalytic performance analyses using monochromatic visible light irradiation show that the quantum efficiency Φ for indigo photooxidation is strongly dependent on the irradiation wavelength, with higher quantum efficiencies being observed at shorter wavelengths (Φ_395nm_ ca. 15%). Recycling tests show that the compound can be employed as homogeneous photooxidation catalyst multiple times without loss of catalytic activity. High turnover numbers (TON ca. 1200) and turnover frequencies up to TOF ca*.* 3.44 min^−1^ are observed, illustrating the practical applicability of the cluster species.

## Introduction

The bottom-up self-assembly of molecular photocatalysts is a well-established method which gives access to materials for which light absorption, catalytic activity and selectivity can be tuned by structural and chemical modifications [1–6]. Prime examples for this approach are molecular metal oxides, so-called polyoxometalates (POMs) [7–8]. POMs are anionic metal oxide clusters formed primarily from early transition metals, mainly vanadium, molybdenum and tungsten [8]. The cluster assembly proceeds in solution by oligo-condensation reactions between reactive fragments, often by using templates to control the final cluster architecture [9–10]. POMs have attracted wide interest from researchers working in chemistry, biology, catalysis, molecular electronics and materials science [8,11–13]. In particular, POMs have been employed as photooxidation catalysts for the oxidation of a wide range of organic substrates such as alcohols, olefins and others [1–3,14]. However, as POMs often only absorb light in the UV range, little is known about the visible-light photocatalytic activity of POMs [5,15]. One means of addressing this challenge is to tune the cluster structure and reactivity by incorporation of a reactive metal site into the cluster shell [15–19]. Using this approach, materials for energy conversion and storage [20–21], homogeneous and heterogeneous catalysis [1,14], biomedical applications [22–24] and nanostructured functional materials [17,25–26] have been developed.

We have recently started systematic studies into the tuning of (photo-)chemical properties of polyoxometalates by selective cluster functionalization with a range of s-block [27–28], p-block [29], d-block [30–31] and f-block [32] metals. Using this approach, we were able to demonstrate that molybdate clusters can be functionalized with single vanadium centres so as to increase their visible-light photoactivity for selective oxidations of alcohols to aldehydes [5–6,31]. Further, we have recently shown that chiral, visible-light driven photooxidation catalysts are accessible in cerium-functionalized vanadate clusters [32]. In previous work with direct relevance to this report, we showed that POM chemistry can be inspired by solid-state photocatalysts when the first molecular analogue of bismuth vanadate (BiVO_4_) photocatalysts was obtained [33–36]. Bismuth vanadate is one of the best-known solid-state visible light photocatalysts and is employed in photochemical and photoelectrochemical visible-light-driven water splitting systems [37–41].

**Scheme 1:**
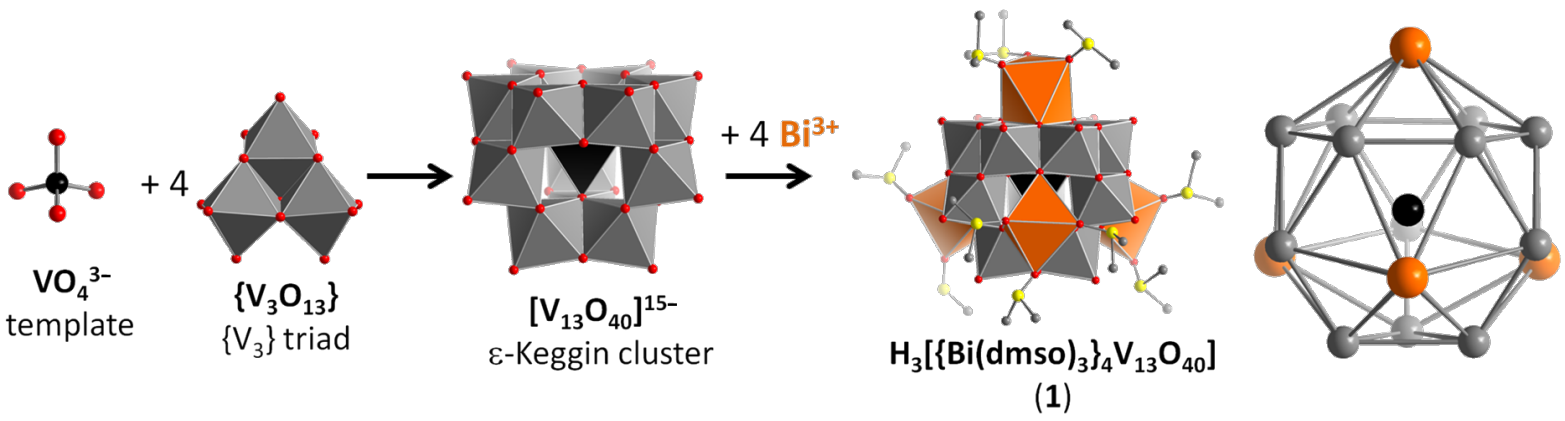
Self-assembly of the H_3_[{Bi(dmso)_3_}_4_V_13_O_40_] cluster **1**. An *ortho*-vanadate (VO_4_^3−^) template allows the assembly of four {V_3_} triads into an ε-Keggin anion ([V_13_O_40_]^15−^). Electrostatic stabilization of the ε-Keggin anion by four Bi(III) centres gives the final, stable cluster H_3_[{Bi(dmso)_3_}_4_V_13_O_40_] (**1**). The metal-only framework of **1** is illustrated on the right. Colour scheme: V (template): black, V (cluster): grey, O: red; Bi: orange, S: yellow; C: dark grey.

At the start of our studies, no molecular bismuth vanadium oxides were known in the literature. We thus developed a synthetic approach to bismuth vanadate clusters based on a recently-established fragmentation-and-reaggregation strategy, see Supporting Information File 1. The route gives general access to metal-functionalized, anion-templated vanadium oxide clusters [5,27–32,42]. Here, this approach was successfully employed and gave the cluster species H_3_[{Bi(dmso)_3_}_4_V_13_O_40_] × ca. 4 DMSO (= **1** × ca. 4 DMSO). **1** is formed spontaneously by reaction of Bi(NO_3_)_3_·5H_2_O and (*n-*Bu_4_N)_3_[H_3_V_10_O_28_] in dimethyl sulfoxide (DMSO) and it was shown that the cluster features both acidic and visible-light photocatalytic activity [29]. To date, to the best of our knowledge, **1** is the only reported example of a molecular bismuth vanadium oxide cluster. Briefly, single-crystal X-ray diffraction showed that the cluster is based on a central *ortho*-vanadate template (VO_4_^3−^). Around this template, four so-called {V_3_} triads ({V_3_O_13_}) are assembled so that each oxygen atom of the VO_4_^3−^ template is linked centrally to one triad. This leads to the formation of a [(VO_4_)V_12_O_36_]^15−^ anion which is the first example of a purely vanadate-based ε-Keggin anion [29,43–45]: As the vanadate anion has a high, destabilizing negative charge (15−), it has previously only been proposed as a transient species with short lifetime using ^51^V NMR spectroscopy [46]. In the present case, the anion is electrostatically stabilized by four bismuth(III) ions which coordinate to binding pockets formed on the cluster surface, see Scheme 1.

Initial photocatalytic studies using the photooxidation of the dye patent blue V as a test reaction showed high activity (quantum efficiency Φ > 7%, TOF ca. 5 min^−1^) and high stability under turnover conditions (TON > 1200) under irradiation with monochromatic visible light (LED light source, λ = 470 nm) [29].

In the present study, we are focusing on the photooxidative performance of the cluster at various wavelengths in the visible region to evaluate cluster application for sunlight-driven oxidation reactions. Further, initial mechanistic catalysis studies are discussed where reactive intermediates and effects of oxygen in the reaction mixture are illustrated.

## Results and Discussion

### Photochemical characterization of the bismuth vanadate cluster **1**

**Figure 1:**
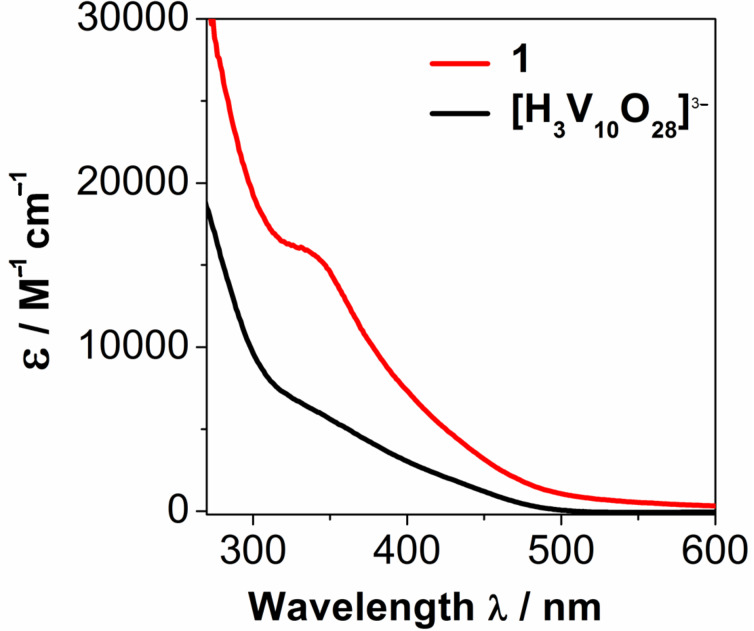
UV–vis spectroscopic data for the bismuth vanadium oxide cluster H_3_[{Bi(dmso)_3_}_4_V_13_O_40_] (**1**, red line). The compound features a near-visible LMCT absorption band with ε_335_ = 15940 M^−1^ cm^−1^. In contrast, the pure vanadium oxide cluster [H_3_V_10_O_28_]^3−^ features significantly reduced visible-light absorption (black line).

UV–vis spectroscopy of **1** shows that the cluster features a prominent ligand to metal charge-transfer (LMCT) absorption band centred at λ = 335 nm with tailing of the absorption band up to λ ≈ 560 nm, making the cluster attractive for visible light absorption. When compared with a prototype pure vanadium oxide cluster such as [H_3_V_10_O_28_]^3−^, the increase in extinction coefficient and the higher visible light absorption are obvious, see Figure 1.

### Photooxidative decomposition of the model pollutant dye indigo

Compound **1** was tested as a homogeneous, visible-light driven photooxidation catalyst for the oxidative degradation of the model pollutant dye indigo (hereafter: Ind). To this end, aliquots of cluster **1** and the dye were mixed in *N*,*N*-dimethylformamide (DMF) solution (typical molar ratio [Ind]:[1] = 5:1). The solutions were subsequently irradiated with monochromatic visible light with wavelengths between 395 nm and 505 nm in order to study the photooxidative performance of the cluster.

**Figure 2:**
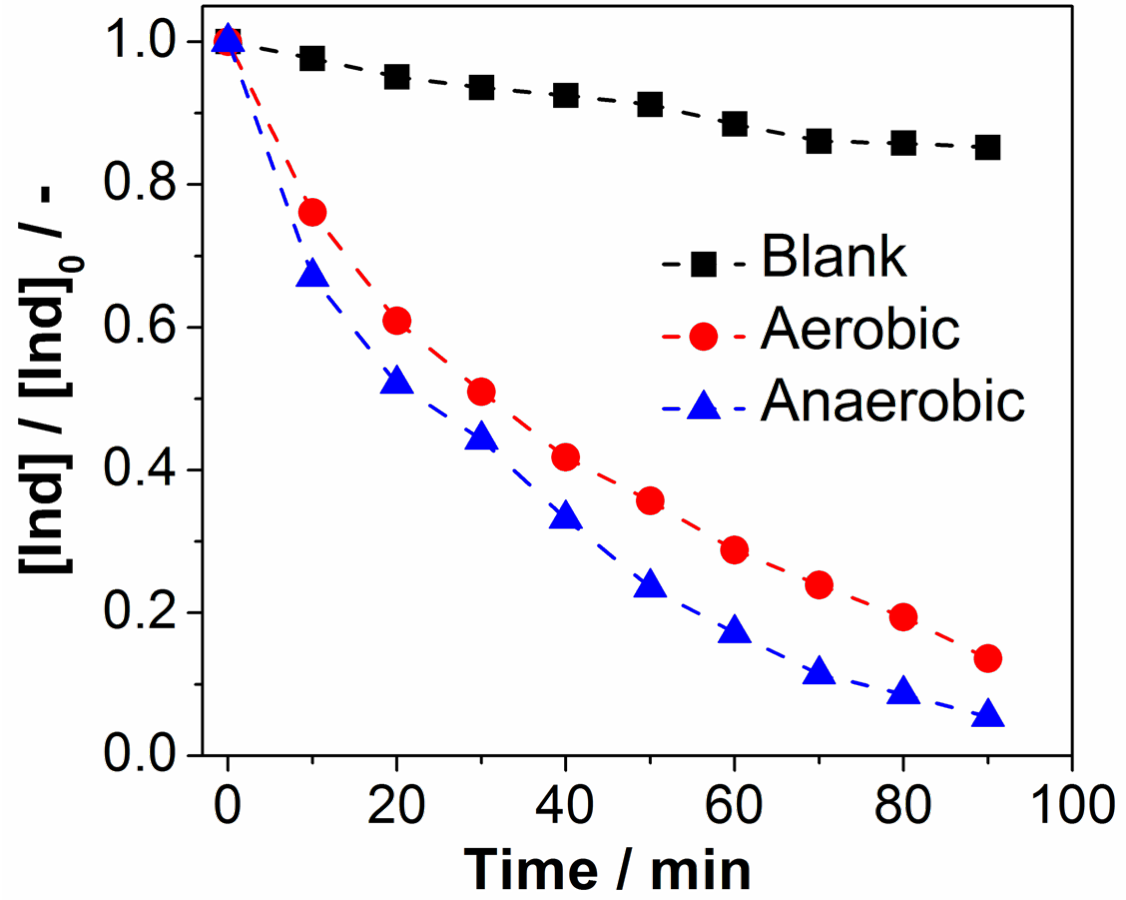
Photooxidative performance of **1** under anaerobic (blue triangles) and aerobic (red circles) conditions in the homogeneous photooxidation of indigo (Ind). Solvent: DMF, light source: monochromatic LED (λ = 430 nm, *P_nominal_* = 3 W), [1] = 1.0 μM, [Ind]_0_ = 5.0 μM).

Standard photooxidative analyses were performed with an irradiation wavelength of λ = 430 nm in DMF. In these screening reactions it was found that **1** performs as an effective visible-light driven photooxidant for the degradation of indigo, see Figure 2.

#### Photooxidative activity of **1**

In detail, it was found that dye degradation under visible light irradiation (here: λ = 430 nm) is significantly enhanced by the presence of photocatalyst **1**. After *t*_irradiation_ = 80 min and under anaerobic conditions, virtually full dye degradation in the presence of **1** is observed ([Ind]/[Ind]_0_ < 3%), see Figure 2. In the absence of any photocatalyst, the dye concentration after an identical irradiation time is [Ind]/[Ind]_0_ ca. 85%, illustrating the visible-light photooxidative activity of **1**. Initial spectroscopic and gas chromatographic analyses suggest that dye degradation is only partial and no full mineralization leading to the formation of CO or CO_2_ was observed.

#### Aerobic vs anaerobic photocatalytic activity of **1**

When comparing the photooxidative performance of **1** in the presence and absence of oxygen, it was found that under anaerobic conditions (solvent degassed with argon for 10 min prior to the experiment), enhanced reaction rates are observed. To quantify the different observed kinetics, observed pseudo-first order rate constants *k*_obs_ were determined for the photooxidation. Comparison of the rate constants clearly demonstrates that anaerobic conditions lead to an approximately 20% increase in reaction rate, suggesting that molecular oxygen interferes with the photooxidation and acts as a quencher for the photoexcited cluster **1** [31,47], see Table 1. Our current hypothesis is that ^3^O_2_ acts as a triplet radical quencher that interacts with the reactive triplet state of the photoexcited cluster molecule by energy and/or electron transfer, resulting in an overall reduced photoreactivity of **1** under aerobic conditions [48]. This hypothesis is currently being investigated using TD-DFT and transient absorption spectroscopy methods.

#### Identification of hydroxyl radicals as intermediates

**Figure 3:**
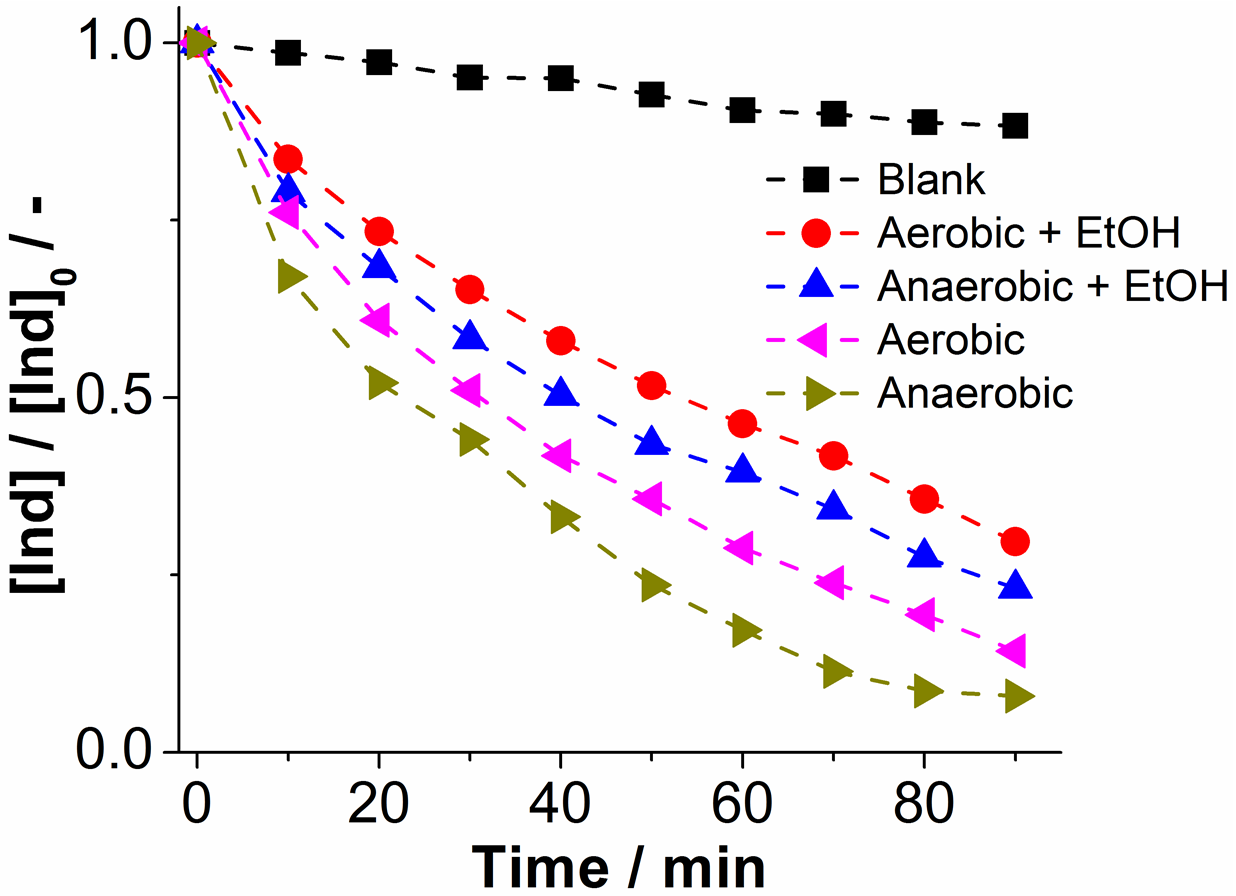
Photooxidative performance of **1** depending on the presence of EtOH under aerobic and anaerobic conditions for the homogeneous photooxidation of indigo (Ind). Solvent: DMF (aerobic/anaerobic), light source: monochromatic LED (λ = 430 nm, *P*_nominal_ = 3 W), [EtOH]:[1] = 50:1.

To gain further insight into the reaction mechanism involved in the photooxidation of indigo by **1**, homogeneous dye degradation was performed in the presence of ethanol in the reaction mixture ([EtOH]:[1] = 50:1). Ethanol is a well-known hydroxyl radical scavenger [49], and the test was performed to demonstrate that hydroxyl radicals are involved as intermediate oxidizing species formed upon irradiation of **1** (see Supporting Information File 1 for proposed mechanism) [5]. If a hydroxyl radical mechanism was present, a significant drop in indigo photooxidation activity would be expected in the presence of EtOH. This behaviour was indeed observed: both under aerobic and anaerobic conditions, significant decrease of reactivity was observed. Quantitative analysis using the observed pseudo-first order rate constant *k*_obs_ gave a decrease in rate constant of ca. 32% (anaerobic) and ca. 44% (aerobic), respectively, see Table 1 and Figure 3. These significant changes are most likely due to an effective scavenging of hydroxyl radical intermediates by ethanol.

**Table 1:** Kinetic parameters for the photooxidation of indigo by compound **1** under aerobic/anaerobic conditions in the presence and absence of EtOH as hydroxyl radical scavenger.

Reaction^a^	*k*_obs_/h^−1^	Standard deviation σ/h^−1^

aerobic	1.41	0.13
anaerobic	1.68	0.20
aerobic + EtOH	0.79	0.15
anaerobic + EtOH	1.14	0.16
blank reference	0.30	0.12

^a^Conditions: Homogeneous reaction, solvent: DMF, irradiation with monochromatic LED light source, λ = 430 nm; [Ind]_0_ = 5.0 μM, [1] = 1.0 μM, [EtOH] = 50 μM.

#### Wavelength-dependent quantum efficiency of **1**

**Figure 4:**
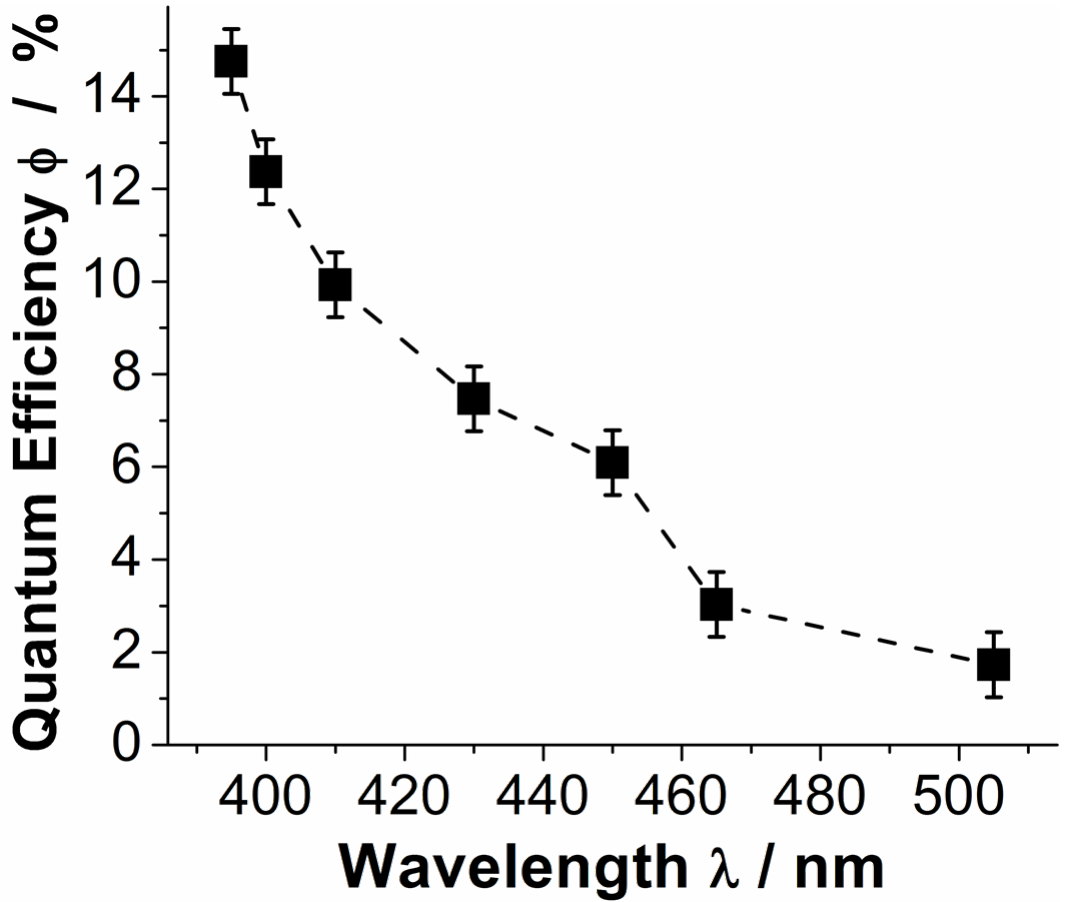
Quantum effiencies Φ for the homogeneous photooxidation of indigo by **1** in the visible range between λ = 395–505 nm. Solvent: DMF (aerobic), molar ratio [Ind]_0_:[1] = 1:1. light source: monochromatic high-power LED, quantum efficiencies were determined using a custom-built setup [49].

In order to gain insight into the wavelength-dependent photoactivity of **1** and to identify the most promising wavelength regions in the visible range, wavelength-dependent quantum efficiency studies for the indigo photooxidation by **1** were performed [50]. As shown in Figure 4, at short wavelengths (λ < 400 nm, quantum efficiencies Φ ca. 15% are observed; in the region between λ = 400–450 nm, quantum efficiencies drop to Φ ca. 12–6%. At wavelengths λ > 450 nm, low quantum efficiencies Φ < 3% are observed, see Figure 4. The data suggest that in the 400–450 nm region, compound **1** might be employed as a homogeneous polyoxometalate photocatalyst with promising efficiencies for this compound class [5].

#### Recyclability of **1**

**Figure 5:**
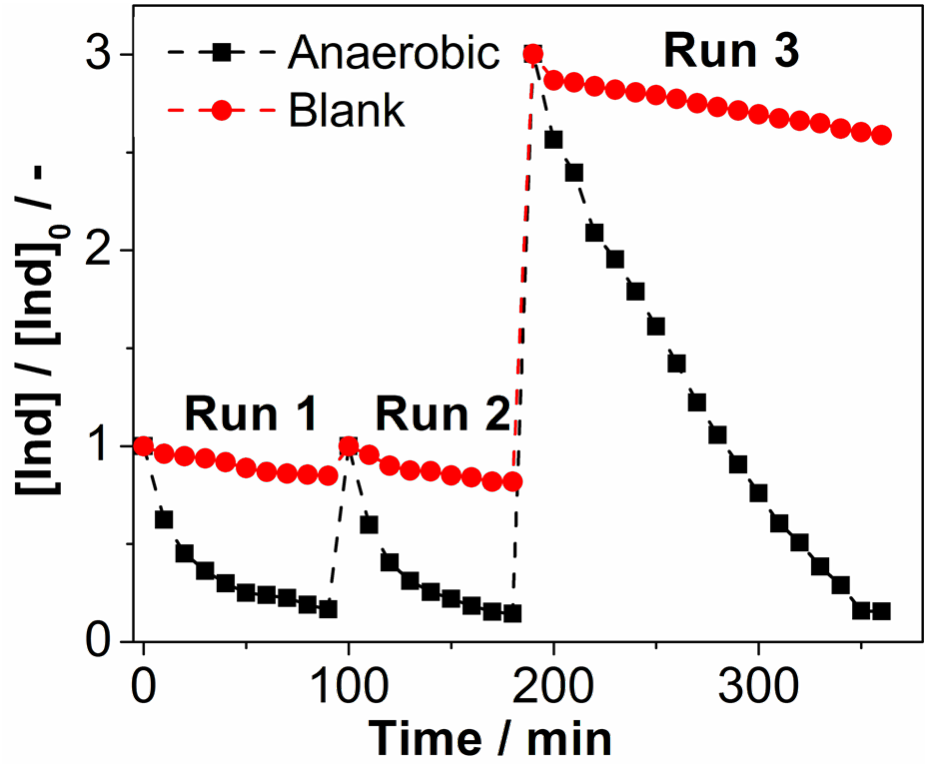
Recyclability of **1** as a homogeneous indigo photooxidation catalyst under anaerobic conditions. Run 1 and run 2 were performed at [Ind]_0_:[1] = 5:1, in run 3, the [Ind]_0_:[1] ratio was increased to 15:1. Solvent: DMF (anaerobic), light source: monochromatic LED (λ = 430 nm, *P*_nominal_ = 3 W).

To demonstrate the long-term stability and recyclability of **1**, three consecutive photooxidative indigo decomposition runs were performed using the same catalyst sample. In the test, a DMF solution containing indigo and **1** ([Ind]_0_:[1] = 5:1) was irradiated under standard conditions (λ = 430 nm) until complete dye decomposition was observed UV–vis spectroscopically (run 1). Using the same sample, the initial indigo concentration was restored by addition of a concentrated indigo stock solution and the photooxidation was repeated to complete indigo degradation (run 2). In run 3, the initial indigo concentration was tripled to demonstrate the photooxidative capacity of the cluster and full dye degradation was observed upon irradiation, see Figure 5. The experimental series demonstrates that the cluster can be employed multiple times and catalyst recycling under photooxidative conditions is indeed possible.

#### Stability of **1** under irradiation

To understand the long-term stability of **1**, long-irradiation runs with high substrate molar ratios up to [Ind]:[1] ca. 1200 were performed. These experiments showed that the cluster compound is capable of photooxidatively decomposing large amounts of the dye, thereby illustrating the chemical robustness of the bismuth vanadium oxide framework. Comparative studies under aerobic and anaerobic conditions showed that the cluster can reach similar turnover numbers (TONs) of ca. 1200, however, higher turnover frequencies for anaerobic conditions (TOF_anaerobic_ ≈ 3.44 min^−1^) were found compared with aerobic conditions (TOF_aerobic_ ≈ 3.01 min^−1^). The findings confirm that under long-term irradiation, the original observation of higher catalytic activity for **1** under anaerobic conditions still holds (see Supporting Information File 1).

## Conclusion

In summary we report the visible-light photocatalytic activity of the first molecular bismuth vanadium oxide cluster, by using the homogeneous photooxidative degradation of the model pollutant indigo as a test reaction. Wavelength-dependent photocatalytic activity is reported and high quantum efficiencies of ca. 15% are observed at the UV–vis border. Practical quantum efficiencies >5% are found up to wavelengths of λ = 450 nm. Recycling studies show that the cluster can be used multiple times without significant loss of activity. Further, initial mechanistic studies show increased cluster reactivity in the absence of oxygen which might act as a quencher for the photoexcited cluster. In addition, we provide initial experimental evidence that the photooxidative mechanism proceeds via the intermediate formation of hydroxyl radicals. Cluster stability with high turnover numbers (ca. 1200) has been demonstrated. Future work will focus on the applications of the cluster for the selective photooxidation of organic substrates (alcohols, olefins). Primary focus will be product selectivity, quantum efficiency as well as long-term catalytic performance.

## Experimental

For experimental, analytical and photocatalytic details, see Supporting Information File 1.

## Supporting Information

File 1 Detailed synthetic, analytic and photocatalytic data.

## References

[1] Hill C L, McCleverty J A, Meyer T J (2003). Polyoxymetalates: Reactivity. Comprehensive Coordination Chemistry II.

[2] Hill C L, Prosser-McCartha C M, Graetzel M, Kalyanasundaram K (1993). Photocatalytic and photoredox properties of polyoxometalate systems. Photosensitization and Photocatalysis using inorganic and organometallic compounds.

[3] Papaconstantinou E (1989). Chem Soc Rev.

[4] Whitesides G M, Mathias J P, Seto C T (1991). Science.

[5] Streb C (2012). Dalton Trans.

[6] Streb C, Kastner K, Tucher J, König B (2013). Polyoxometalates in Photocatalysis. Chemical Photocatalysis.

[7] Long D-L, Burkholder E, Cronin L (2007). Chem Soc Rev.

[8] Pope M T (1983). Heteropoly and isopoly oxometalates.

[9] Pope M T, Müller A (1991). Angew Chem, Int Ed Engl.

[10] Pope M T (1992). Nature.

[R11] 11Kortz, U.; Liu, T., guest Eds. Polyoxometalates (Cluster Issue). *Eur. J. Inorg. Chem. ***2013,** 1556–1967.

[12] Cronin L, Müller A (2012). Chem Soc Rev.

[13] Hill C L (1998). Chem Rev.

[14] Hill C L (2007). J Mol Catal A.

[15] Argitis P, Papaconstantinou E (1986). Inorg Chem.

[16] Geletii Y V, Botar B, Kögerler P, Hillesheim D A, Musaev D G, Hill C L (2008). Angew Chem, Int Ed.

[17] Long D-L, Tsunashima R, Cronin L (2010). Angew Chem, Int Ed.

[18] Muradov N, T-Raissi A (2006). J Sol Energy Eng.

[19] Gao G, Li F, Xu L, Liu X, Yang Y (2008). J Am Chem Soc.

[20] Lv H, Geletii Y V, Zhao C, Vickers J W, Zhu G, Luo Z, Song J, Lian T, Musaev D G, Hill C L (2012). Chem Soc Rev.

[21] Sartorel A, Bonchio M, Campagna S, Scandola F (2013). Chem Soc Rev.

[22] Hasenknopf B (2005). Front Biosci, Landmark Ed.

[23] Aureliano M, Fraqueza G, Ohlin C A (2013). Dalton Trans.

[24] Judd D A, Nettles J H, Nevins N, Snyder J P, Liotta D C, Tang J, Ermolieff J, Schinazi R F, Hill C L (2001). J Am Chem Soc.

[25] Kurth D G (2008). Sci Technol Adv Mater.

[26] Streb C, Tsunashima R, MacLaren D A, McGlone T, Akutagawa T, Nakamura T, Scandurra A, Pignataro B, Gadegaard N, Cronin L (2009). Angew Chem, Int Ed.

[27] Kastner K, Puscher B, Streb C (2013). Chem Commun.

[28] Kastner K, Streb C (2013). CrystEngComm.

[29] Tucher J, Nye L C, Ivanovic-Burmazovic I, Notarnicola A, Streb C (2012). Chem–Eur J.

[30] Forster J, Rösner B, Fink R H, Nye L C, Ivanovic-Burmazovic I, Kastner K, Tucher J, Streb C (2013). Chem Sci.

[31] Tucher J, Wu Y, Nye L C, Ivanovic-Burmazovic I, Khusniyarov M M, Streb C (2012). Dalton Trans.

[32] Seliverstov A, Streb C (2014). Chem Commun.

[33] Li G, Zhang D, Yu J C (2008). Chem Mater.

[34] Kudo A, Omori K, Kato H (1999). J Am Chem Soc.

[35] Jia Q, Iwashina K, Kudo A (2012). Proc Natl Acad Sci U S A.

[36] Li R, Zhang F, Wang D, Yang J, Li M, Zhu J, Zhou X, Han H, Li C (2013). Nat Commun.

[37] Saito R, Miseki Y, Sayama K (2012). Chem Commun.

[38] Seabold J A, Choi K-S (2012). J Am Chem Soc.

[39] Zhong D K, Choi S, Gamelin D R (2011). J Am Chem Soc.

[40] Kudo A (2001). J Ceram Soc Jpn.

[41] Abdi F F, Han L, Smets A H M, Zeman M, Dam B, van de Krol R (2013). Nat Commun.

[42] Forster J, Rösner B, Khusniyarov M M, Streb C (2011). Chem Commun.

[43] Yamase T, Makino H, Naruke H, San José Wéry A M (2000). Chem Lett.

[44] Dolbecq A, Mialane P, Lisnard L, Marrot J, Sécheresse F (2003). Chem–Eur J.

[45] Dolbecq A, Mellot-Draznieks C, Mialane P, Marrot R, Férey G, Sécheresse F (2005). Eur J Inorg Chem.

[46] Pettersson L, Andersson I, Howarth O W (1992). Inorg Chem.

[47] Tanielian C, Schweitzer C, Seghrouchni R, Esch M, Mechin R (2003). Photochem Photobiol Sci.

[48] Grewer C, Brauer H-D (1994). J Phys Chem.

[49] Billany M R, Khatib K, Gordon M, Sugden J K (1996). Int J Pharm.

[50] Megerle U, Lechner R, König B, Riedle E (2010). Photochem Photobiol Sci.

