# Supplementary material for: Visible light photooxidative performance of a high-nuclearity molecular bismuth vanadium oxide cluster
Source: Beilstein J Nanotechnol. 2014 May 26;5:711–6. doi: 10.3762/bjnano.5.83 (PMC4077371; doi:10.3762/bjnano.5.83)
Supplement: File 1 — Detailed synthetic, analytic and photocatalytic data. [file Beilstein_J_Nanotechnol-05-711-s001.pdf]

Supporting Information

for

Visible light photooxidative performance of a high-nuclearity molecular bismuth vanadium oxide cluster

Johannes Tucher, and Carsten Streb*[§]

Address: Ulm University, Institute of Inorganic Chemistry I, Albert-Einstein-Allee 11, 89081 Ulm, Germany.

Email: carsten.streb@uni-ulm.de;

*Corresponding author

[§]web: www.strebgroup.net

Detailed synthetic, analytic and photocatalytic data

1. Instrumentation

X-ray diffraction: Single-crystal X-ray diffraction studies were performed on a Nonius Kappa CCD Single-crystal X-ray diffractometer equipped with a graphite monochromator using MoKα radiation (wavelength λ(Mo-K$_\alpha$) = 0.71073 Å).

UV–vis spectroscopy: UV–vis spectroscopy was performed on a Shimadzu UV-2401PC spectrophotometer, Varian Cary 50 spectrophotometer or Varian Cary 5G spectrophotometer equipped with a Peltier thermostat.

FTIR spectroscopy: FTIR spectroscopy was performed on a Shimadzu FT-IR-8400S spectrometer. Samples were prepared as KBr pellets. Signals are given as wavenumbers in cm^{-1} using the following abbreviations: vs = very strong, s = strong, m = medium, w = weak and b = broad.

Elemental analysis: Elemental analysis was performed on a Euro Vector Euro EA 3000 Elemental Analyzer.

General remarks: All chemicals were purchased from Sigma Aldrich or ACROS and were of reagent grade. The chemicals were used without further purification unless stated otherwise.

2. Synthesis of $H_3[Bi_4(dmso)_{12}V_{13}O_{40}]$ × ca. 4 DMSO

General assembly mechanism

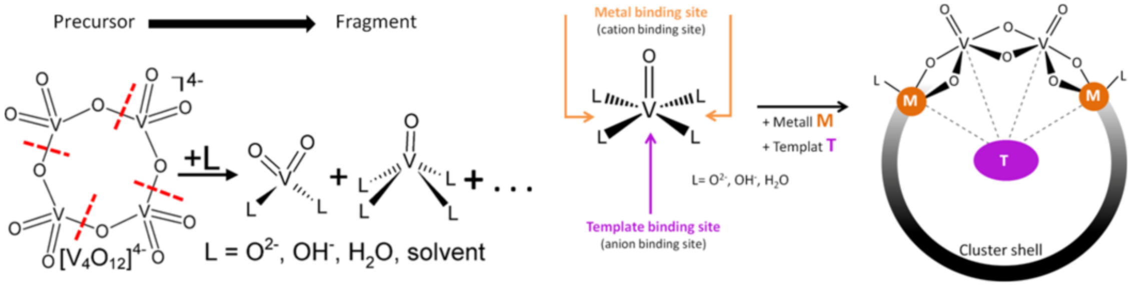

Figure S1: General assembly mechanism of metal-functionalized vanadium oxide clusters: fragmentation of a labile precursor (here: $[V_4O_{12}]^{4-}$ leads to reactive fragments. Coordination of the desired metal to the reactive fragment and subsequent cluster assembly driven by a template leads to the template-stabilized, metal functionalized cluster.

The synthesis is a modified version of the original synthesis [1].

For the synthesis of $H_3[Bi_4(dmso)_{12}V_{13}O_{40}]$ × ca. 4 DMSO (**1**), $(n\text{-}Bu_4N)_3[H_3V_{10}O_{28}]$ (0.250 g, 148.12 μmol) and $Bi(NO_3)_3 \times 5H_2O$ (0.144 g, 296.86 μmol) are suspended in dimethyl sulfoxide (15 mL). 0.15 mL deionized water is added and the orange suspension is heated to 80 °C to give a clear, orange solution. Then, 5 µL of a 1.5 M aqueous $n\text{-}Bu_4NOH$ solution is added and a deep red, homogeneous solution is obtained. The reaction was vigorously stirred and heated to 80 °C for one hour after which the clear,

deep red solution was cooled to rt. The pure compound was obtained as single crystals by diffusion of ethyl acetate into the mother liquor. The crystalline product was filtered off, washed with diethyl ether and dried in a desiccator. Yield: 0.230 g (67.8 μmol, 91.3% based on Bi)

Elemental analysis for $C_{32}H_{99}Bi_4O_{56}S_{16}V_{13}$ (Mw: 3391.3 g/mol) in wt % (calcd.): C 11.11 (11.33), H 2.90 (2.94), S 13.67 (15.13); Bi 24.25 (24.65); V: 19.50 (19.52). The elemental analysis indicates the loss of approximately 2 DMSO molecules during the drying process.

Characteristic IR bands (in cm^{-1}): 3001 (w), 2915 (w), 2359 (w), 1628 (m), 1414 (m), 1317 (w), 993 (m), 949 (s), 804 (s), 608 (vs).

3. UV–vis spectroscopy

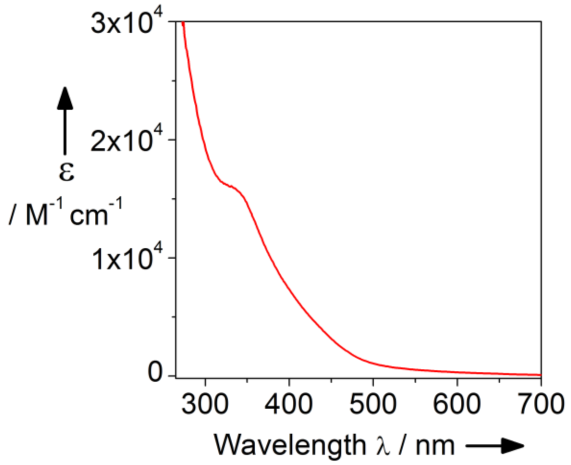

Figure S2: UV–vis spectroscopic analysis of **1** in *N*,*N*-dimethylformamide (DMF), showing the tailing of the LMCT transitions into the visible region.

4. Photooxidation reactions

The following mechanism is reported in the literature for the hydroxyl radical mediated substrate photooxidation by polyoxometalate clusters [2-4].

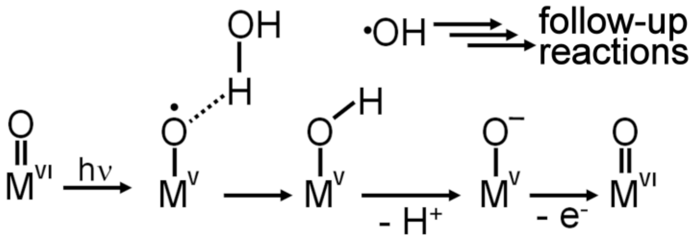

Figure S3: Hydroxyl radical-mediated substrate photooxidation by polyoxometalate clusters. Irradiation of a O→M ligand to metal charge-transfer band leads to a charge-separated state and formation of an oxo-centred radical on the cluster shell. Reaction of the oxo radical with pre-associated water leads to hydrogen-atom abstraction and hydroxyl radical formation. The hydroxyl radical dissociates from the cluster shell and undergoes follow-up oxidative reactions.

Photooxidations were performed by dissolving **1** and indigo in *N*,*N*-dimethyl formamide (DMF). Clear, homogeneous solutions were obtained. The solutions were irradiated using LED light sources in custom-built irradiation setups. Standard light source was a LED with λ = 430 nm, $P_{nominal}$ = 3 W. Standard molar ratios were [Ind]:[**1**] = 5:1. Deaerated experiments were performed by de-gassing the samples with Argon for 10 min prior to the experiment. As blank references, identical samples as described above without added photocatalyst **1** were used.

Cluster re-oxidation is required for a photocatalytic process to take place. To this end, a terminal electron acceptor is required. Currently, we hypothesize that the electrons are transferred from the cluster to molecular oxygen and (under de-aereated conditions) to organic fragments/degradation products of the indigo dye used. The exact details are currently under investigation using time-resolved transient UV–vis spectroscopy.

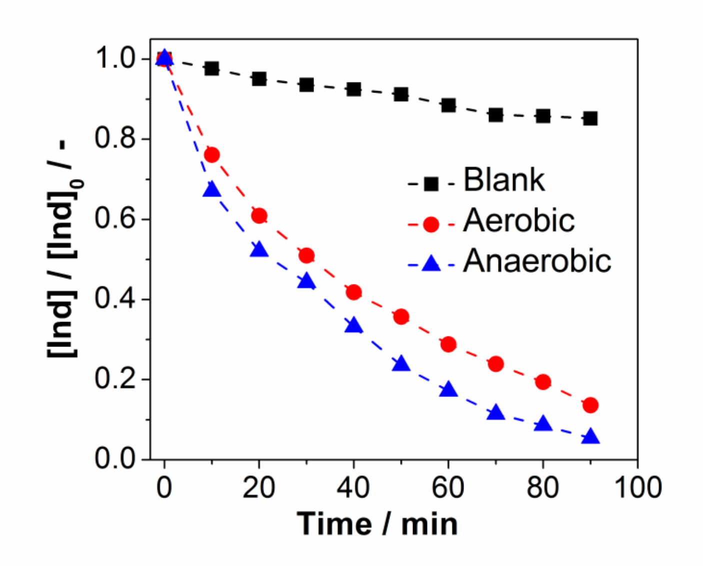

Figure S4: Photocatalytic indigo decomposition catalyzed by **1**. Conditions: LED-irradiation, λ = 430 nm, $P_{nominal}$ = 3 W; *N*,*N*-dimethylformamide; the anaerobic sample was de-gassed with Ar for 10 min prior to the experiment. $[Ind]_0$ = 5.0 µM; [**1**] = 1.0 µM. Ind = indigo.

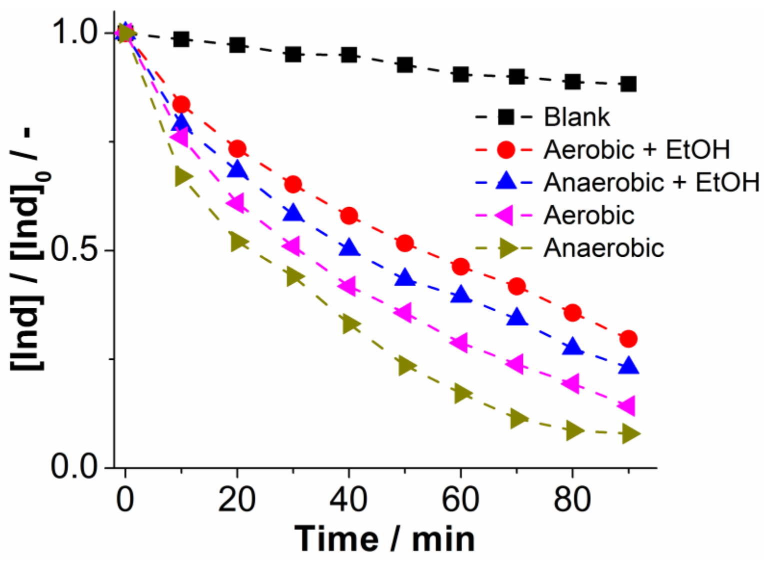

Figure S5: Photocatalytic indigo decomposition catalyzed by **1** in the presence or absence of ethanol (EtOH) under aerobic and anaerobic conditions. Conditions: LED-irradiation, λ_{max} = 430 nm, $P_{nominal}$ = 3 W; solvent: *N*,*N*-dimethylformamide (DMF, aerobic and anaerobic). $[Ind]_0$ = 5.0 µM; [**1**] = 1.0 µM, [EtOH] = 50 µM. Ind = indigo.

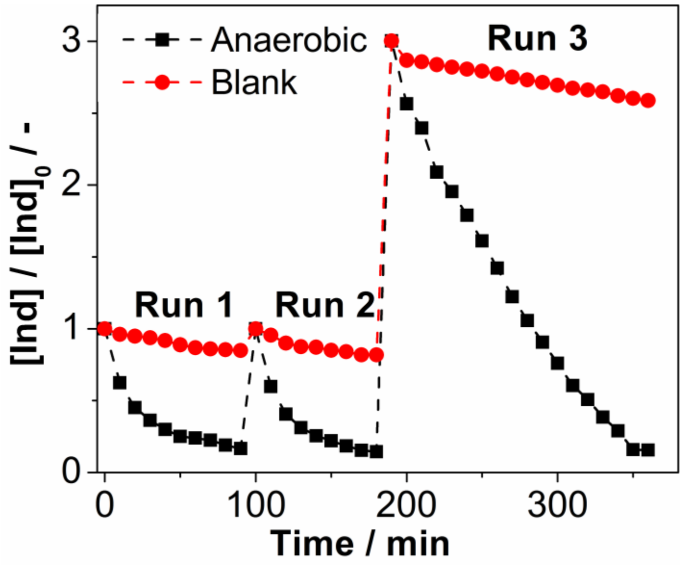

Figure S6: Recyclability of the bismuth vanadium oxide photocatalyst **1** demonstrated in three consecutive runs where Ind was decomposed under monochromatic visible-light irradiation (LED-irradiation, λ = 430 nm, $P_{nominal}$ = 3 W). Conditions: solvent: *N*,*N*-dimethyl formamide (DMF, anaerobic) $[Ind]_0$ = 5.0 µM; [**1**] = 1.0 µM. In Run 3, the dye concentration was tripled $[Ind]_0$ = 15 µM to demonstrate the system capacity.

Determination of the turnover number TON and turnover frequency TOF

Turnover number and turnover frequency were determined under the following reaction conditions: $[Ind]_0$ = 5.0 µM; [**1**] = 4.17 nM; irradiation source: 3 W LED-light source (λ_{max} = 430 nm); solvent: an- and aerobic *N*,*N*-dimethyl formamide and anaerobic sample was degassed with Ar for 10 min prior to the experiment. After $t_{irradiation}$ = 345 min (anaerobic) and $t_{irradiation}$ = 399 min (aerobic) indigo decomposition was complete, giving *TON* = 1200 and $TOF_{aerobic}$ = 3.01 min^{-1}, $TOF_{anaerobic}$ = 3.44 min^{-1}, see Figure S7.

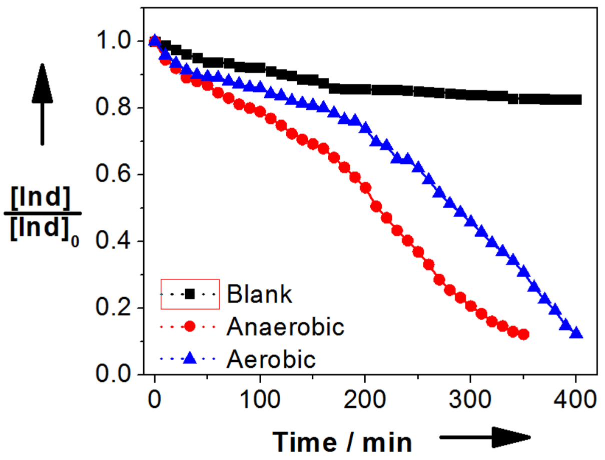

Figure S7: UV–vis spectroscopic determination of the turnover number and turnover frequency for the photochemical indigo degradation by **1**. Conditions: LED-irradiation (λ = 430 nm, $P_{nominal}$ = 3 W), solvent: *N*,*N*-dimethyl formamide (DMF, aerobic and anaerobic). ([**1**] = 4.17 nM, $[Ind]_0$ = 5 μM).

5. Quantum efficiency determination

Quantum efficiencies were determined using a custom-built irradiation and quantum efficiency determination setup reported by König, Riedle et al. [5].

Table S1: Quantum efficiency determination for the indigo photooxidation by **1**

λ / nm	Quantum efficiency / %
395	14.75
400	12.37
410	9.93
430	7.47
450	6.09
465	3.03
505	1.73

6. References

1. Tucher, J.; Nye, L. C.; Ivanovic-Burmazovic, I.; Notarnicola, A.; Streb, C. *Chem.–Eur. J.* **2012,** *18,* 10949–10953. doi:10.1002/chem.201200404
2. Hill, C. L. Polyoxymetalates: Reactivity. In *Comprehensive Coordination Chemistry II*; McCleverty, J. A.; Meyer, T. J. Eds. Elsevier Ltd. 2003, Vol. 4, pp679–759. doi:10.1016/B0-08-043748-6/03036-X
3. Papaconstantinou, E. *Chem. Soc. Rev.* **1989,** *18,* 1–31. doi:10.1039/cs9891800001
4. Seliverstov, A.; Streb, C. *Chem. Commun.* **2014,** *50,* 1827–1829. doi:10.1039/c3cc48834a
5. Megerle, U.; Lechner, R.; König, B.; Riedle, E. *Photochem. Photobiol. Sci.* **2010,** *9,* 1400–1406. doi:10.1039/c0pp00195c
